# Understanding Potential Heavy Metal Contamination, Absorption, Translocation and Accumulation in Rice and Human Health Risks

**DOI:** 10.3390/plants10061070

**Published:** 2021-05-26

**Authors:** Zuliana Zakaria, Nur Syahirah Zulkafflee, Nurul Adillah Mohd Redzuan, Jinap Selamat, Mohd Razi Ismail, Sarva Mangala Praveena, Gergely Tóth, Ahmad Faizal Abdull Razis

**Affiliations:** 1 Department of Food Science, Faculty of Food Science and Technology, Universiti Putra Malaysia, UPM Serdang 43400, Selangor, Malaysia; zulianaz@moh.gov.my (Z.Z.); nursyahirahzulkafflee@gmail.com (N.S.Z.); dillaredzuan94@gmail.com (N.A.M.R.); sjinap@gmail.com (J.S.); 2 Laboratory of Food Safety and Food Integrity, Institute of Tropical Agriculture and Food Security, Universiti Putra Malaysia, UPM Serdang 43400, Selangor, Malaysia; smpraveena@upm.edu.my; 3 Laboratory of Climate-Smart Food Crop Production, Institute of Tropical Agriculture and Food Security, Universiti Putra Malaysia, UPM Serdang 43400, Selangor, Malaysia; razi@upm.edu.my; 4 Department of Environmental and Occupational Health, Faculty of Medicine and Health Sciences, Universiti Putra Malaysia, UPM Serdang 43400, Selangor, Malaysia; 5 Department of Soil Science and Environmental Informatics, Georgikon Faculty, University of Pannonia, H-8360 Keszthely, Hungary; toth.gergely@agrar.mta.hu; 6 Natural Medicines and Products Research Laboratory, Institute of Bioscience, Universiti Putra Malaysia, UPM Serdang 43400, Selangor, Malaysia

**Keywords:** heavy metals, rice, paddy soil, health risk assessment

## Abstract

Rice is a worldwide staple food and heavy metal contamination is often reported in rice production. Heavy metal can originate from natural sources or be present through anthropogenic contamination. Therefore, this review summarizes the current status of heavy metal contamination in paddy soil and plants, highlighting the mechanism of uptake, bioaccumulation, and health risk assessment. A scoping search employing Google Scholar, Science Direct, Research Gate, Scopus, and Wiley Online was carried out to build up the review using the following keywords: heavy metals, absorption, translocation, accumulation, uptake, biotransformation, rice, and human risk with no restrictions being placed on the year of study. Cadmium (Cd), arsenic (As), and lead (Pb) have been identified as the most prevalent metals in rice cultivation. Mining and irrigation activities are primary sources, but chemical fertilizer and pesticide usage also contribute to heavy metal contamination of paddy soil worldwide. Further to their adverse effect on the paddy ecosystem by reducing the soil fertility and grain yield, heavy metal contamination represents a risk to human health. An in-depth discussion is further offered on health risk assessments by quantitative measurement to identify potential risk towards heavy metal exposure via rice consumption, which consisted of in vitro digestion models through a vital ingestion portion of rice.

## 1. Introduction

Because there is an increasing trend of population growth and demand of rice consumption, the use of an escalating number of pesticides by producers, in order to protect their crop, may influence the level of heavy metals in soils [1,2]. Heavy metal pollution on a paddy field has recently been of great concern as an environmental pollutant due to of its bioaccumulation in the environment and non-biodegradable properties [3]. Heavy metals have been commonly discussed as a potential pollutant in rice and it is classified as one of the major important toxic substances, due to its high potential risk to the ecosystem and human health [3].

The International Agency for Research on Cancer (IARC) (2012) [4] has classified arsenic (As), cadmium (Cd), chromium (Cr), and nickel (Ni) as group 1 carcinogen, since long-term exposure leads to increased risk of various type of cancer, including disruptions in tumor suppressor gene expression, damage repair processes, and enzymatic activities that are concerned in metabolism through oxidative damage [5]. By nature, heavy metals can be divided into two forms, which are organic and inorganic [6]. For example, inorganic As is more harmful than organic form due to the pentavalent inorganic compound of As that solubilizes in water to the weak acid form and produce arsenate [7] that affects people by inducing ground water contamination [8]. Heavy metals in the form of organic pollutant may gradually degrade into less harmful components through chemical or biological processes [9,10]. Environmental contamination of the biosphere with heavy metals have caused intensive agricultural and other anthropogenic activities and posed critical problems for safe use of agricultural land [11]. Agricultural soils are potentially contaminated with essential and nonessential heavy metals through existing agriculture practice with arbitrarily use of agrochemicals, such as pesticide and fertilizers, along with mechanical cultivation that change the speciation and the mobility of heavy metals [12,13].

In subsistence farms of Asia, there is a clear verification of the association between Cd poisoning and human renal dysfunction [14] and, certainly, rice has been recognized as one of the prime sources of Cd and Pb to human intake especially in Japan [15,16,17,18]. Plant organs have different abilities to adsorb, translocate, and accumulate heavy metals, and the speed and magnitude of these processes vary between plant species and cultivars [19,20,21,22,23,24]. Other than that, the adsorption and accumulation of heavy metals in soil is driven by soil properties, such as pH, and the concentration of organic matter [25]. The direct transfer of heavy metals to human body from plant parts of interest is rice grain, which is an edible part for consumption that may cause a threat to human health [25].

Because heavy metals are proclaimed to amass in living organisms, especially toxic metals, such as As, Cd, and Pb, health risk assessments are conducted on the basis of heavy metal concentration found in paddy plants and soils to evaluate the potential health risk [26]. The heavy metal taken up from plants may transfer into the food chain and adversely affect human health, depending on their carcinogenicity [26]. People are potentially exposed to toxic metals and metalloids from rice, especially those who take it as staple food for daily energy requirement. The tolerable daily intake (TDI) for Cd, Hg, and As, which are highly toxic to human exposure, are recommended to be 1.0 µg/kg body weight (bw)/day, 0.57 µg/kg (bw)/day, and 2 to 7 µg/kg (bw)/day, respectively [27].

Understanding the soil-metals uptake mechanisms in food crop is essential for devising the effective remediation process. In order to achieve this goal, this review initially describes the mechanism of heavy metal uptake in paddy plants and soils and their contributing factors with respect to bioaccumulation. Impressively, this review tries to provide an overview on the human risk assessment in relation to non-carcinogenic and carcinogenic risk. In this regard, it also covers information regarding *in vitro* digestion models that can be conducted to determine the bioaccessible form of heavy metals.

## 2. Heavy Metal Contamination in Paddy Plants and Soils

Table 1 shows the summary of heavy metal concentration in paddy plants and soil in selected areas of different countries. A study conducted by Singh et al. [28] at Ramgarh Lake, India, found that the Zn, Cr, Cu, and Pb contents are higher in the cropped soils rice plants as compared to different parts of rice plants, except for Cd and As, which is due to their adsorptive nature in soil [29]. Meanwhile, the metals in rice plants are found to be mostly accumulated in the roots, rather than in other parts, such as stalk and grain sub-samples. Generally, As is found to be of the highest metal uptake by the roots in experimental sites, whereas, in the grains, the As concentration were less than in the roots and straw of paddy crops. Liu et al. [30] suggested that roots function as barrier for metal translocation by protecting stem and grain parts from metal contamination. Looi et al. [31] also have reported that concentration of As was found to be the highest (4.62 mg/kg) in roots due to the presence of iron plague on root surface that is highly associated with As accumulation, as shown in Table 1.

Meanwhile, at East Coast of India, specifically in East Coast Road (ECR), Tamil Nadu, Satpathy et al. [32] reported that Zn was of the highest and cadmium (Cd) was of the lowest concentrations of heavy metals in paddy soils. For different parts of paddy plants, the finding is almost similar to Singh et al. [28], where most of the metals accumulated more in the roots than in other parts, which are shoots and grains. The element Zn which is known as a micronutrient, is of the highest metal uptake in roots, followed by Pb, Cr, Cu, and Cd. Most metals in paddy plants, such as Fe, Mn, Zn, and Cu, were found extensively and they are micronutrients that were required in various enzyme activities and play significant roles in photosynthesis and growth of the plants [33,34]. The concentration of Cd in shoots was greater than roots and grain, since Cd was easily taken up by plants and transported to different parts, although there is no beneficial effects and it is nonessential for plants and animals [35]. Besides, this metal also reduced the photosynthesis and uptake of nutrient when the plants are exposed [36].

In China, paddy soils that were collected from three areas of Hunan Province during harvest season in 2013 were severely polluted with Cd with a total mean of 1.4 mg/kg [37], which are significantly higher as compared to the Chinese environmental quality standard (0.3 mg/kg) and the corresponding background values of Hunan (0.098 mg/kg). The variation in Cd concentration was associated with the geology area and resulting from human mining activities [37]. Similarly, other elements, such as As and Pb, showed a slightly higher concentration in comparison with standard and background values due to agriculture activities, including the application of various pesticides and fertilizers [38,39]. Moreover, studies that were conducted on the paddy soil from Nanxun and Suxian counties revealed that Zn and Pb concentration had the largest mean value, both exceeding 100 mg/kg [40,41].

The excessive usage of pesticide and herbicide could result in the accumulation of Zn, Pb, As, and Cu in the topsoil of agricultural fields [42]. Hence, the long-term application of fertilizer and pesticides may influence the level of heavy metal in soils [43,44]. The element of As in rice grain from Gangneung, South Korea, and Zhejiang Province, China has accumulated by 0.13 mg/kg and 0.08 mg/kg, respectively [45,46]. It was reported that As can be easily and largely accumulate by all cereal types due to its high bioavailability under reduced soil conditions [47]. Moreover, rice crop has great efficiency in assimilating As into grain as compared to other staple cereal crops [48]. Zeng et al. [37] reported that the concentration of As in brown rice may not only be affected from soil concentration, it but could also by other factors, such as physical-chemical properties of the growing soil, including the equilibrium pH that affect heavy metal sorption and desorption on soil components. According to Du et al. [49], most of the soils in Hunan are acidic and they may enhance the mobility of heavy metals from soil to rice.

In Malaysia, Khairiah et al. [50] and Looi et al. [31] studied the heavy metal accumulation of paddy cultivation in Kedah, whereas the study in Sabah was conducted by Yap et al. [51], Aziz et al. [52], and Payus et al. [53] in three different areas. Based on Table 1, the concentration of Cd in rice grain and other parts of paddy plants, such as leaf, stem, and roots by Khairiah et al. [50] was lower when compared to Yap et al. [51]. The authors reported that the application of pesticides and fertilizers in Langkawi did not influence the increase of Cd content in paddy plant. The level of Cr in paddy root, as reported by Payus et al. [53], was greater than shoot part. which includes main stem and plant above 10 cm from root postulated due to occurrence of redox reaction in plants that caused the movement of chromium from root to the shoot part. Moreover, chromium (III) can also react with carboxylic functional groups (-COOH) in plants. This kind of reaction can distract the translocation of the metal elements from root to shoot and, thus, result in low Cr concentration in shoot parts [54]. Most of the metals (Pb, Cd, Cr, and Cu) concentrate in roots of paddy, except for Zn, which is highly accumulated in the stems of paddy and soils. The accumulation of Zn in plants is due to the absorption of the metal by roots from the plants that are enclosed with soil [55]. The plants require the Zn element as an important nutrient in order to synthesize proteins, hormone growth, and reproductive processes of plants. Nonetheless, the excessive level of Zn in plants, which is more than 200 mg/kg, will cause toxicity, resulting in stunted root growth and undersized leaves in plants [55].

**Table 1:** Summary of heavy metals concentration in paddy plants and soil in selected areas of different countries.

Area of Study	Sample(s)	Mean Concentration of Heavy Metals (mg/kg)						References
		Cd	As	Pb	Cr	Cu	Zn	
Nanxun County, China	Rice grain	0.01	-	-	-	2.49	14.28	Zhao et al. [40]
	Soil	0.21	-	33.2	-	31.06	106.82	
Ramgarh Lake, Gorakhpur, UP, India	Roots	6.16	22.77	7.09	2.93	2.09	2.24	Singh et al. [28]
	Rice grain	0.01	0.08	0.54	0.09	-	-	
	Rice straw	0.64	0.87	1.88	-	-	-	
	Soil	0.05	7	23	62.5	24	73	
Kompipinan, Papar district, Sabah, Malaysia	Roots	0.38	-	7.7	5.46	4.94	16.08	Payus et al. [53]
	Stem	0.11	-	0.04	3.26	0.38	29.6	
	Leaf	0.11	-	0.26	4.34	0.71	12.4	
	Grain	0.13	-	2.06	4.12	0.74	12.75	
	Soil	0.32	-	8.03	4.16	6.62	13.89	
Gangneung, South of Korea	Rice	0.01	0.13	0.01	-	-	-	Choi et al. [45]
	Rice seed	0.001	0.22	0.1	-	-	-	
	Rice straw	0.04	0.81	0.03	-	-	-	
	Rice root	0.05	2.07	5.29	-	-	-	
	Fertilizer	0.01	1.22	5.61	-	-	-	
	Soil	0.1	0.54	5.93	-	-	-	
Kubang Pasu, Kedah, Malaysia	Soil	0.2	0.6	3.72	2.3	-	-	Looi et al. [31]
	Root	0.29	4.62	1.35	0.57	-	-	
	Stem	0.06	0.02	0.07	0	-	-	
	Grain	0.01	0.06	0.21	0.04	-	-	
Hunan Province, China	Soil	1.4	16.8	51.4	27.2	-	-	Zeng et al. [37]
	Brown rice	0.31	0.34	0.02	0.106	-	-	
		**Mean concentration of heavy metals (mg/kg)**						**References**
**Area of study**	**Sample(s)**	**Cd**	**As**	**Pb**	**Cr**	**Cu**	**Zn**	
Suxian County, South China	Soil	2.94	64.51	179.63	-	46.62	-	Song et al. [41]
Ranau Valley, Sabah, Malaysia	Soil	0.45	3.54	-	3360.56	154.83	229.98	Aziz et al. [52]
	Rice grain	0.54	0.05	-	1.61	2.61	37.48	
Kota Marudu, Sabah, Malaysia	Rice	0.18	-	ND	1.34	0.31	0.69	Yap et al. [51]
	Husk	0.18	-	ND	0.73	0.19	0.52	
	Leaf	0.2	-	ND	1.02	1.24	1.21	
	Stem	0.24	-	ND	0.71	1.53	0.68	
	Root	0.19	-	1.57	1.86	9.25	2.31	
	Soil	0.78	-	ND	2.08	ND	21.09	
Zhejiang Province, China	Rice	0.04	0.08	0.06	-	-	-	Huang et al. [46]
East Coast Road (ECR), India	Soil	0.02–0.60	-	5.30–19.80	1.30–7.80	0.03–5.40	3.80–33.8	Satpathy et al. [32]
	Shoot	0.20–0.30	-	0.30–1.20	0.40–0.90	0.04–0.30	2.30–6.00	
	Root	0.11–0.20	-	3.60–5.30	0.60–1.70	0.20–0.50	4.70–16.90	
	Grain	0.02–0.05	-	0.01–1.00	0.10–0.60	0.10–0.30	3.20–7.20	
Langkawi, Kedah, Malaysia	Rice	0.02–0.04	-	0.06–0.08	-	0.04–0.08	0.18–0.22	Khairiah et al. [50]
	Leaf	0.01–0.02	-	0.06–0.09	-	0.20–0.52	3.71–7.17	
	Stem	0.01–0.02	-	0.04–0.08	-	0.07–0.24	0.78–1.08	
	Root	0.02	-	0.10–1.06	-	0.08–0.34	0.77–1.16	
	Soils	0.01–0.03	-	0.28–0.51	-	0.14–0.20	0.23–0.47	

Note: -, Not included in analysis; ND, Not Detected.

## 3. Mechanism of Heavy Metals Uptake in Plants

Many researchers have investigated contaminant uptake and its mechanism by plants. It might be useful to regulate the contributing factors to enhance the mechanism of plant uptake. With reference to Usman et al. [56], the plants normally play its role both as “accumulators” and “excluders”. Accumulators sustain even with concentrating contaminants in their aerial parts. The plants biotransform or biodegrade the contaminants into inactive forms in their tissues. The excluders, in principle, limit the uptake of contaminant into their biomass.

Plants have developed very specific and highly efficient mechanisms to attain crucial micronutrients from the environment, albeit when existing at low ppm and ppb levels. The plant roots, which are assisted by plant-producing chelating agents and plant-inducing pH changes and redox reactions, can dissolve and absorb micronutrients from down levels in the soil, even from partly not soluble sediments. Plants have also changed very explicit mechanisms to translocate and store micronutrients in their parts. The same mechanisms are also embraced in the uptake, translocation, and storage of heavy metals, where the nature of the chemical property’s mimics those of essential elements. Therefore, mechanisms of micronutrient uptake are of great attention to phytoremediation [57].

The array of known transport mechanisms set in the plant cell plasma membrane included in ion uptake and translocation involve (i) co- and anti-transporters (proteins that utilize the electrochemical gradients that are produced by ATPases to initiate the active uptake of ions), (ii) proton pumps (ATPases that occupy energy and produce electrochemical gradients), and (iii) channels (proteins that ease the transport of ions into the cell). A range of ions is likely will be taken up by each transport mechanism. The ionic species interaction during several heavy metal contaminants uptake is the primary problem in the mechanism of uptake. Following roots uptake, the translocation of contaminants into shoots is required, since the harvest of biomass from root is generally not achievable. There were lack of information concerning which forms of metal ions are translocated from the roots to the shoots [58].

Heavy metals uptake mechanisms by plants are expected to be strictly controlled. Plants, in general, do not accumulate trace elements over their metabolic needs. Such settings are trivial in the range of 10 to 15 ppm of most trace elements suitable for most needs [59]. The exemptions are plants that are classified as “hyperaccumulator”, which can absorb toxic metal ions at a concentration in the thousands of ppm. In addition, other matter is the form of which toxic metal ions are accumulated in the plants, specifically in hyperaccumulator, and how these plants are resistant to metal toxicity. In principle, manifold mechanisms are involved; including the storage in the plant’s vacuole seems to be a major one [60].

Evapotranspiration is a term used to explain the process of water evaporating from plant leaves into plant roots. The water serves as a pump to disseminate nutrients absorbed to the plant roots and shoots. The original soil was leaved undisturbed in order to remove contamination from roots to shoots. The plants that execute a shoot-to-a metal-concentration ratio that is greater than one are called “hyperaccumulators”, and these plants are normally used in phytoextraction strategies. Otherwise, the plants with shoot-to-root ratio less than one are categorized as non-accumulating plants. Theoretically, toxic environment should be flourished with hyperaccumulators, where it requires less maintenance and generates high biomass [61].

Heavy metals, like Cd, Zn, Co, Mn, Ni, and Pb, can be accumulated by metal accumulating plant species concentrated up to 100 or 1000 times higher than the excluder (non-accumulator) plants. Typically, microorganisms living in the rhizosphere, like bacteria and fungi, may promote mobilizing metal ions and increase the bioavailable fraction [62,63].

**Figure 1:**
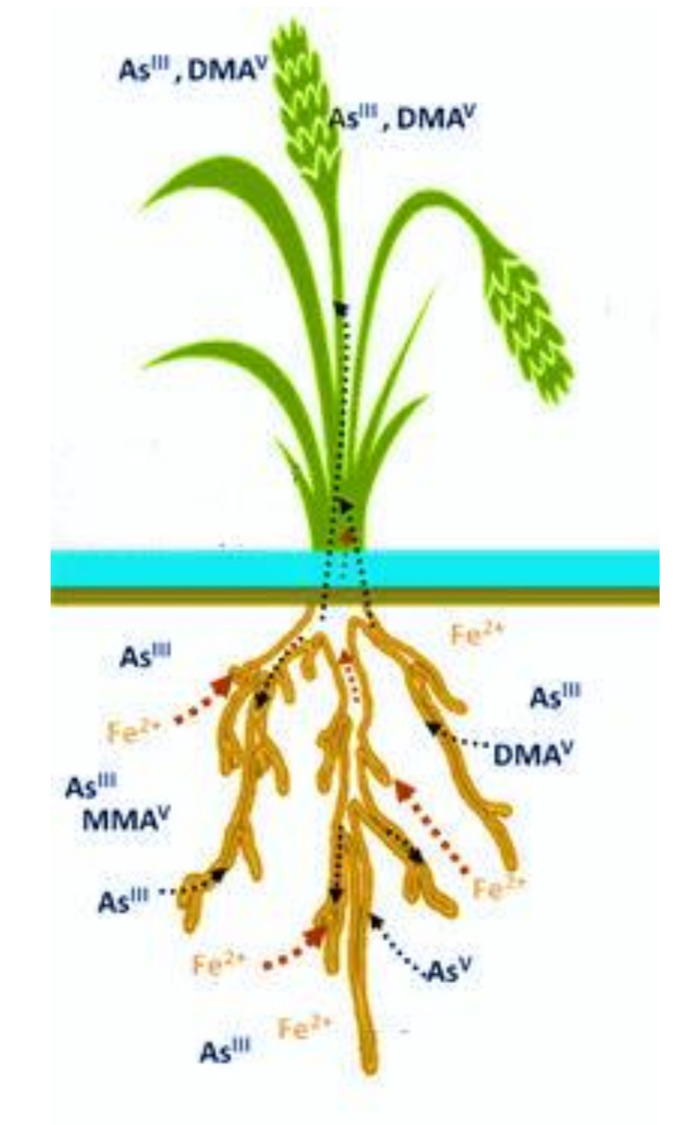
Uptake mechanism of arsenic in paddy plants. Adapted from Zhao et al. [55]. Copyright permission granted by Copyright Clearance Center.

Figure 1 showed the proposed mechanism of As uptake in paddy plants by Zhao et al. [64]. The As speciation in rice grain is dominated by dimethylarsinic acid (DMA^V^) and inorganic As (iAs), which are majorly composed of arsenite, As (III). The total iAs is generally reported by the sum of two species through the conversion between As (III) and arsenate, As (V), which may occur during some extraction procedures [65]. In the biotransformation process, these iAs are converted to methylated arsenicals by enzymes, which are the biomarker of chronic arsenic exposure and the end metabolites [65]. Verbruggen et al. [66] reported that the phosphate transporters in As translocation manage the influx of arsenate, whereas the arsenite is taken up via aquaporin nodulin 26–like intrinsic proteins. In the xylem sap of plants, inorganic As (V) and As (III) are the major arsenicals found [67]. A very small proportion of As is translocated to shoot tissue (leaf), where similar reduction and sequestration mechanisms exist and, via the phloem, some of the total As content ceases in the vacuoles and other parts of edible tissue, such as rice grain [67]. Different rice varieties and rice genotypes have shown significant differences in accumulating As at its higher concentration [68]. This might be partly due to the surface characteristics of rhizosphere, which also play an important role in iron plague formation of reddish-brown coating on the root surface [68].

Besides As, the irrigation water in paddy field often also causes loads of Cd in soil. It accumulates in grains following four major transport processes, namely (1) the uptake of Cd by root, (2) root-to-shoot translocation by xylem flow, (3) redirection of nodes, and (4) remobilization from leaves [69]. When the element Cd binds to cysteine-rich protein, such as metallothionein, their concentrations are increased to 3,000-fold [65]. Phytochelatins (PCS) has been classified as class III metallothioneins and Cd is known as the strongest inducer for their biosynthesis [70]. The Cd element is able to form complexes with PCS and is transported to vacuoles [71]. PCS-metal complexes possibly form a more complex aggregation in vacuole by isolating toxic metals from various metal sensitive enzymes in plant cell cytoplasm [71].

### 3.1. Factors Affecting the Uptake Mechanisms

**Figure 2:**
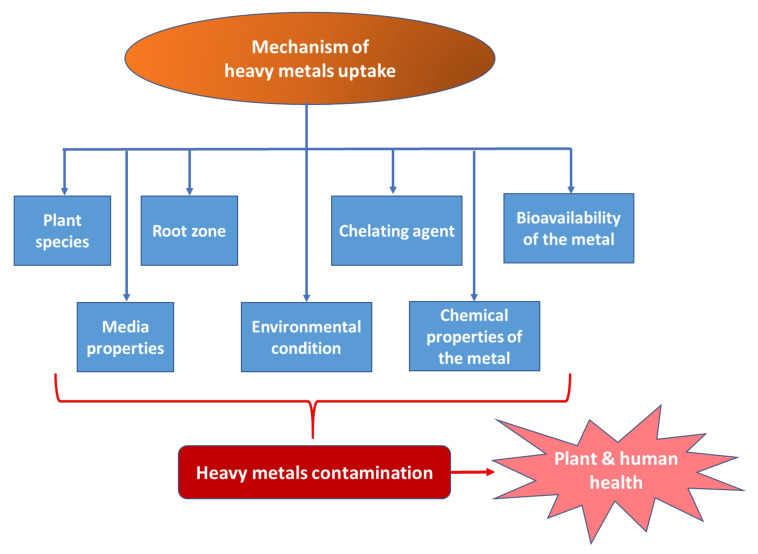
Factors which are affecting the uptake mechanisms of heavy metals.

There are several factors that can affect the uptake mechanism of heavy metals, as shown in Figure 2. By having knowledge about these factors, the uptake performance by plant can be greatly improved.

#### 3.1.1. The Plant Species

Plant species or varieties play important roles in the phytoremediation process, which includes phytofiltration, phytoextraction, phytostabilization, phytovolatilation, phytodegradation, and rhizodegradation [72,73]. The uptake of a compound is affected by plant species characteristics [73]. The success of the phytoremediation depends upon the identification of suitable plant species that hyperaccumulate heavy metals and produce large amounts of biomass while using established crop production and management practices [74]. Various plants have different responses toward various heavy metals exposure. Some plants are sensitive, whereas others have a high tolerance to several heavy metals. As a result of plant-metal interaction, some plants accumulate heavy metals from soil and, thus, their growth and development declines. Nevertheless, some plants have a high tolerance and they maintain their growth and development below heavy metals stress [73].

#### 3.1.2. The Properties of Medium

Remediation enhancement via pH adjustment, the addition of chelators, and fertilizers employed in agronomical practices may affect metals uptake [75]. As such, to reduce the amount of lead absorbed by plants, the pH of the soils is adjusted to a level of 6.5 to 7.0. This is due to lead absorption being affected by the pH, organic matter, and phosphorus content of the soil [76].

#### 3.1.3. The Root Zone

The root zone is of special interest of the plant tissues in phytoremediation. Th root is able to absorb, store, or metabolize contaminants inside the plant tissue. Another phytoremediation mechanism is the degradation of contaminants in the soil by plant enzymes and rhizospheric microorganisms that exudes from the plant’s roots via phytodegradation and rhizodegradation, respectively [73]. As a result of morphological adaptation to drought stress, the root diameter of a plant is increased, and root elongation is reduced as a response to less permeability of the dried soil [77].

#### 3.1.4. Vegetative Uptake

Environmental conditions will affect the vegetative uptake [78], where the temperature affects the growth substances and, subsequently, root length. There are differences in terms of the structure between the root under field conditions and greenhouse conditions [79]. Contaminant-specific hyperaccumulator will determine the success of phytoremediation, specifically the phytoextraction [80]. The key to proving the applicability of phytoremediation is to understand the mass balance analyses and the metabolic fate of pollutants in plants [81]. The bioavailability of the metal in the water phase will determine the metal uptake by plants. This is supported by the retention time and interaction of the metal with other elements and substances in the water. Moreover, the metal-soil bound, pH, redox potential, and organic matter content are other factors that affect the tendency of the metal to exist in ionic and plant-available form. The ability of plants to lower the pH and oxygenate the sediment will affect the soil condition and metal content [82]. Chelating agents and micronutrients as biodegradable physicochemical factors are also added to increase the bioavailability of the heavy metals [83].

#### 3.1.5. The Chelating Agent

Increasing the bioavailability of heavy metals through the addition of biodegradable physicochemical factors, such as chelating agents and micronutrients, can influence the uptake of heavy metals by the energy crops. Other than that, microbial community in and around the plant will also stimulate the heavy metal uptake capacity. Consequently, the remediation periods will become shorter and less expensive. The use of synthetic chelating agent in heavy metal contaminated soil could promote the leaching of the contaminants into the soil. Therefore, the proper usage of the agent has to be taken in consideration [84]. For example, the use of a chelating agent may be required in alkaline soils since the bioavailability of heavy metals in soils decreases above pH 5.5–6. The metal translocation in plant tissue as well as the overall phytoextraction performance has been improved when exposing the plants to EDTA for longer period (two weeks) [85]. The bioavailability of metals is affected by citrate and oxalate that are exuded by plant roots. The enhancement of phytoextraction of soil-polluting heavy metals is aided by chelate-assisted phytoremediation chelating agents, such as NTA and EDTA. The presence of a ligand affects the bio uptake of heavy metals through the formation of metal-ligand complexes and it changes the potential to leach metals below the root zone [86].

### 3.2. Bioaccumulation Factor (BAF)

The soil-to-rice transfer factor or bioaccumulation factor (BAF) is an index for evaluating the potential of a metal transfer from soil to plant [87]. BAF is calculated for each rice sample by the ratio of the element concentration in the grain to that in the corresponding soil to quantify the bioaccumulation effect of rice towards the heavy metal uptake from the soils [12,88,89] (Equation (1)). The BAF is measured as:

(1) BAF = Cr/Cs

where Cr and Cs represent the heavy metal concentrations in rice grain and soils, respectively, based on the basis of dry weight (*w*/*w*) in mg/kg. If the BAF is ≤ 1, then the value denotes that the plant is able to absorb the heavy metals, but do not accumulate, whereas BAF > 1 means that the heavy metals accumulate in plants [28,32]. According to Ma et al. [90] and Cluis [91], BAF is further classified as hyperaccumulators and excluder to the samples that accumulate metals > 1 mg kg^−1^, and < 1, respectively. The toxic effect of heavy metals from excluder plant is restricted to the roots, which is then detoxified, and the aerial parts of plants remain more or less unaffected. Meanwhile, the hyperaccumulator plants are able to accumulate without phytotoxicity symptoms in their aboveground parts, even if exposed to high concentration of heavy metals [92,93].

Table 2 summarizes BAF values in paddy plants from different areas by several studies. The toxic metals, such as mercury (Hg) and cadmium (Cd), were found to be higher than other studied metals in BAF values by Singh et al. [28] and Satpathy et al. [32], respectively. However, the BAF values for both toxic metals were less than one, which indicates that the plants only absorb the heavy metals and are not metal accumulators. Hence, the local inhabitants of respective areas have low exposure to heavy metal, since the soil-to-plant transfer is one of the key components of human exposure to metals via food chain [94]. From Table 2, the BAF values for Mn (1.88) from Neeratanaphan et al. [95] and all metals (Zn, Cd, Cr, Pb, and Cu) from Payus et al. [53] were more than 1, which indicated that *Oryza sativa* is a hyper accumulator plant with high potential to absorb metals from the soil [96]. The accumulation, uptake, and phytotoxicity may also vary, depending on cultivars used. Previously, Xie and Huang [97] discovered that there were significant differences for the uptake and accumulation of As in different parts of plants among the 11 rice cultivars planted in As-polluted paddy fields. However, there were no significant differences found by Xie at al. [98] for BAF of Pb, Cd, Cr, As, and Cu among the conventional rice, two-line hybrid rice, and three-line hybrid rice, which indicate that different rice varieties from the same soil background have no relation to the bioaccumulation ability of heavy metals for rice.

### 3.3. Translocation Factor (TF)

The translocation factor (TF) is known as an indicator of heavy metal accumulation in plants or mobility of heavy metals in the soil and it also quantifies the differences in the bioavailability of metal to plant [100]. According to Barman et al. [101] and Gupta et al. [102], the TF or mobilization ratio was calculated to evaluate relative translocation of metals from soil to other parts, such as root, shoot, or grain, of the plant species, as follows (Equation (2)):

(2) TF = Cs/Cr

where Cs represents heavy metal concentration in plants’ shoot and Cr represents heavy metal concentration in plants’ soil or root [28]. The value of TF greater than 1 showed that the paddy plant is able to hyperaccumulate from roots to shoots, according to Rezvani and Zaefarian [103]. By evaluating TF, the bioavailability of heavy metals in investigated soils could be revealed. The higher the TF values are, the more mobile or available the metals are [104,105,106].

Table 3 summarizes TF values in paddy parts from different areas by various studies. The soil-to-root translocation in rice plant by Singh et al. [28] and Rahimi et al. [99] found that the Cd value is more than 1, which indicates that rice root accumulated high quantities of Cd^2+^ when grown in polluted areas [107]. Because Cd^2+^ was substantiated to be more bioavailable than other heavy metals, hence, it resulted in higher biological absorption coefficient for Cd [28,108]. There are certain species of plants that are capable of removing heavy metals from the soil and ground water through absorption and accumulation by roots, or precipitation within root zone [109]. Other than that, the application of genetic engineering on plants, such as transgenic rice, may significantly enhance plant abilities to uptake, translocate, and transform heavy metals, including limiting their toxicity [110]. The genetic modification in rice by enhancing drought tolerance can also potentially affect non-target organisms or nutrient mobilization, as discovered by Jeong et al. [111], due to overexpressing the OsNAC10 gene in roots that cause pleiotropic effects on other genes that play a role in nutrient mobilization. Thus, this modification might enhance the uptake of the metals, resulting in elevated metal concentration in the aboveground plant parts, which can affect essential nutrients and causes oxidative stress.

### 3.4. Enrichment Factor (EF)

There is a limited study on the enrichment factor (EF) specifically for paddy plants and soil. EF was calculated in order to derive the degree of soil contamination and heavy metal accumulations in soil and in plants growing on contaminated site with respect to soil and plants growing on uncontaminated soil [112]. EF was calculated by the following equation (Equation (3)):

(3) $$\mathrm{EF}\;=\;\frac{\mathrm{Concentration}\;\mathrm{of}\;\mathrm{metals}\;\mathrm{in}\;\mathrm{soil}\;\mathrm{or}\;\mathrm{plant}\;\mathrm{parts}\;\mathrm{at}\;\mathrm{contaminated}\;\mathrm{site}}{\mathrm{Concentration}\;\mathrm{of}\;\mathrm{metals}\;\mathrm{in}\;\mathrm{soil}\;\mathrm{or}\;\mathrm{plant}\;\mathrm{parts}\;\mathrm{at}\;\mathrm{uncontaminated}\;\mathrm{site}}$$

Satpathy et al. [32] found that all metals (Cu, Cd, Zn, Cr, Mn, and Pb) showed EF values that were greater than 1, which indicates that the samples have relatively high potential to uptake metals from the soil [96]. In addition, the high availability and metal distribution in contaminated soils eventually cause metal accumulation in plant species grown in soil to increase [102,112]. Similarly, the EF values of eight metals (Cr, Cd, Mn, Pb, As, Zn, Hg, and Cu) in experimental soil, root, stem, and grain parts of paddy were greater than 1 [28]. Barman and Bhargava [113] reported that the EF value for edible parts, such as rice grain, is an important criterion for the selection of suitable crop species, which can be selected for cultivation in a field having an elevated level of metal contamination or receiving industrial effluent.

## 4. Human Diseases Associated with Heavy Metal Contamination in Rice

Rice is a major crop globally and it is especially important in Asia [114,115]. Therefore, the information on rice consumption with toxic metal contamination and its health-related issue is of paramount importance. Cd, As, and Pb are among the major contaminant in rice in Asia, where they have been ranked as the most hazardous substances according to the criteria of frequency of occurrence in the environment, toxicity, and potential exposure to humans [116]. The increased risks of all-cancer mortality were reported due to long-term environmental exposure to Cd [117].

Studies that were conducted at a few locations in (a) Japan [118,119,120], (b) China [49], and (c) Thailand [121] similarly found the relationship between human Cd disease and the survival of rice farmers. Cadmium exposure was found to be related to the consumption of rice and presented a high risk to local farmers in Japan particularly the female farmers over 70 years of age had decreased function of renal tubular [119]. Locally produced rice in Japan was found to be contaminated with Cd and those people who had Cd poisoning from rice consumption suffered spinal and leg pain with other complications, including anaemia, coughing, and kidney failure, which primarily caused death [118]. This phenomenon is also known as the ‘itai-itai’ disease, which generally related to environmental pollution due to local mining activities in Japan. The heavy metal contamination and its accumulation in China has also become a serious and major environmental problem because of the continuous industrialization and urbanization [122,123]. The Hunan Province, for instance, which provides the main area for rice production, had a food safety issue in regards to the discovery of “Cd rice” [49], which resulted in a high incidence of malignant tumors among the local population [117,124]. Besides Japan and China, rice grains grown in Thailand, specifically at Mae Sot district of Tak Province, have elevated levels of Cd resulting in higher amounts of urinary Cd as compared to those living in other districts [121]. In 2010, Swaddiwudhipong and colleagues [125] have reported a greater excretion rate of urinary total protein, which reflects that Cd exposure might cause severe tubular damage and/or glomerular permeability to larger proteins. Therefore, cadmium-exposed persons should be screened for other medical conditions, such as diabetes and urinary stones, found to be related to proteinuria that lead to neuropathy.

Rice, together with other food and drinking water, may be a source of considerable combined exposure to inorganic arsenic. Inorganic arsenic, which is more toxic, is known to cause several diseases and cancers of different parts of the body, including the skin, cardiovascular system, and reproductive systems [126], and even diabetes through epigenetic mechanisms, as reported by several studies in South Asia [63]. Studies by Jayasumana et al. [127] revealed that the usage of phosphate fertilizer is a main source of As in areas that are affected with chronic kidney disease in Sri Lanka.

Moreover, among the hazardous heavy metals, Pb has been listed as “the chemical of great concern” by the new European REACH regulations, and it was known as the second most harmful pollutant following arsenic [128]. High levels of Pb in rice grain employed quality issues and adverse health implications to human liver, endocrine, and reproductive system [129].

## 5. Health Risk Assessment (HRA) of Heavy Metals in Rice

Health risk assessments (HRA) are generally focused on target populations by evaluating their potential health effects when exposed to certain toxic elements. HRA includes non-carcinogenic and carcinogenic risk assessments through three exposure pathways, which are ingestion, dermal contact, and inhalation, which have been recognized as important tools for identifying health risks in human activities and providing risk evidence for decision makers [130].

Indeed, rice consumption was considered to be the major pathway of human exposure to heavy metals [46]. The HRA that was conducted by Man et al. [131], Zota et al. [132], and Qu et al. [133] found that children are highly susceptible to heavy metal pollution as compared to adults due to their physiological and behavioral characteristics. The toxicity of metals in terms of toxicological effects may vary in each person. One way to predict their bioaccumulation and transformation is to develop a mathematical model for HRA when associated with heavy metal contamination, especially in soil [134].

There are various mathematical models for risk assessments, such as health risk index (HRI), target cancer risk (TCR), hazard index (HI), and target hazard quotient (THQ), which have been used to assess the carcinogenicity of the particular heavy metals present, especially in food and drinks. Table 4 tabulates the mathematical models for risk assessment on heavy metal exposure on rice consumption. Uncertainties might occur when conducting risk assessments from natural variability in an individual’s response, variability in toxicants concentration, in measurement or parameters estimation, a lack of precise knowledge, and data scarcity [135,136]. Therefore, deterministic or stochastic approaches can be used as health risk assessment models. Monte Carlo Simulation (MCS) is an example of a stochastic approach, and it has been widely used in risk assessment. Djahed et al. [137] used MCS for both non-carcinogenic and carcinogenic risk assessments on rice consumption in Iran to specify the uncertainty by analyzing and controlling the existing uncertainty in the input parameters.

### 5.1. Exposure Estimates

Chronic daily intake (CDI) can be used to evaluate heavy metal exposure from soil through inhalation, dermal contact, and ingestion [122,138], whereas average daily dose (ADD) or estimated daily intake (EDI) can be measured for exposure from rice grain.

#### 5.1.1. Chronic Daily Intake (CDI)

Heavy metals potentially accumulate in the human body through the food chain and, thus, constitute serious health threats [139]. There are three pathways that are involved in the estimation of the direct exposure to heavy metal via soil, which are: (i) the inhalation of particulates emitted from the soil, (ii) dermal contact with the soil, and (iii) incidental ingestion of the soil [122]. The CDI of these three exposure pathways can be defined employing U.S Environment Protection Agency (USEPA) methodology [140]. The exposure pathways can be measured using the following equation:

(4) $${\mathrm{CDI}}_{\mathrm{Inhalation}}\;=\;\frac{{\mathrm{PM}}_{10}{\;\times \;M}_{\mathrm{PM}}{\;\times \;\mathrm{ET}\;\times \;\mathrm{IR}}_{\mathrm{air}}\;\times \mathrm{EF}\;\times \mathrm{ED}}{\mathrm{BW}\;\times \mathrm{AT}\;\times \mathrm{PEF}\;}$$

(5) $${\mathrm{CDI}}_{\mathrm{Dermal}}\;=\;\frac{C_{\mathrm{soil}\;}\times \;\mathrm{SA}\;\times \;\mathrm{PE}\;\times \;\mathrm{AF}\;\times \;\mathrm{ABS}\;\times \;\mathrm{ED}}{{\mathrm{BW}\;\times \mathrm{AT}\;\times \;10}^{6}\;}$$

(6) $${\mathrm{CDI}}_{\mathrm{Ingestion}}\;=\;\frac{C_{\mathrm{soil}\;}{\times \;\mathrm{IR}}_{\mathrm{air}}\;\times \;\mathrm{EF}\;\times \;\mathrm{ED}}{{\mathrm{BW}\;\times \;\mathrm{AT}\;\times \;10}^{6}\;}$$

where PM_10_ is the ambient particulate matter in the area of study (mg/m^3^); M_PM_ is the heavy metal concentration of airborne particulate matter, which is assumed to be equal to C_soil_, where dust is derived from the soils [141]; ET is the exposure time (hours/day); IR_air_ represents the inhalation rate of air (m^3^/day); EF is the exposure frequency (days/year); ED is the exposure duration (year); C_soil_ is the concentration of heavy metals in soil (mg/kg); SA is the skin surface area for soil contact (cm^2^/day); FE is the fraction of dermal exposure ratio to the soil; AF is the soil adherence factor (mg/cm); ABS is the fraction of applied dose absorbed across the skin; and, 10^6^ is the conversion factor, from kg to mg.

Based on the findings from Liang et al. [138], soil ingestion was the main contributor in all of the soil exposure pathways, whereby the soil pathways are reduced in the order of ingestion > dermal absorption > inhalation. Similarly, Hu et al. [122] and Xiao et al. [142] also found the same trend of CDI values for ingestion, dermal absorption, and inhalation. Therefore, soil ingestion was the most significant contributor to the total health risk of inhabitants.

#### 5.1.2. Average Daily Dose (ADD) and Estimated Daily Intake (EDI)

ADD or EDI is a parameter used to calculate the oral exposure dosage during a specific period by expressing it as a daily dose per unit body weight [143]. The equation of ADD or EDI in Equations (7) and (8) was formulated as:

(7) ADD = (C × IR × EF × ED)/(BW × AT)

(8) EDI = (C × CON)/BW

where C is the heavy metal concentration in rice grain (mg/kg); Con is the average daily consumption of rice in the region (kg/day); and, BW is the average body weight of people in the region (kg) [144]. IR, ED, EF, and AT represent the ingestion rate, exposure duration, exposure frequency, and averaging time, respectively [41,145]. The ADD or EDI of metals rely on the concentration of metal in food and daily amount of food consumed [41,95,143]. The EDI of heavy metals via rice ingestion reported by Neeratanaphan et al. [95] was higher than the previous study conducted by Huang et al. [146], which indicates that the rice consumption among local inhabitants in Khong Chai district of the Kalasin Province in Thailand is probably exposed to heavy metal contamination. The average daily intake of rice among adults and children living around Huludao Zinc Plant in China was 389.2 and 198.4 g/person/day, respectively [87]. Fan et al. [143] reported that the ADD value for Cd is much higher than other heavy metals and suggested a heavy daily intake of Cd from rice for adult from three main areas of mines (Liuyang, Hengyang, Loudi) of Hunan Province, China. The perennial intake of contaminated rice crops is likely to induce adverse health effects from heavy metal exposures.

**Table 4:** Mathematical models for risk assessment on heavy metal exposure. Adapted from EPA [147].

Model of Risk Assessment	Mathematical Equation	Description of Equation
Health Risk Index (HRI)	$\sum \frac{n\;(\mathrm{Cn}\;\times \;\mathrm{Dn})}{\mathrm{RfD}-\mathrm{Bw}}$ or $\frac{\mathrm{EDI}}{\mathrm{RfD}}$	Cn = Heavy metal concentrations in samples (mg/kg) Dn = Daily intake of samples (mg/person/day) RfD = Oral reference dose (mg/kg/day) Bw = Body weight for children and adults (kg) EDI = Estimated Daily Intake (mg/kg/day)
Hazard Index (HI)	$\overset{n}{\underset{i=1}{\sum }}\mathrm{HRI}$ or ∑HQ	HRI = Hazard risk index HQ = Hazard Quotients
Hazard Quotient (HQ)	$\frac{\mathrm{ADI}}{\mathrm{RfD}}$	ADI = Average Daily Intake (mg/kg.day) RfD = Oral reference dose (mg/kg/day)
Target Hazard Quotients (THQ)	$\frac{\mathrm{MC}\;\times \;\mathrm{FI}\;\times \;\mathrm{EFr}\;\times \;\mathrm{ED}}{\mathrm{RfD}\;\times \;\mathrm{Bw}\;\times \;\mathrm{AT}\;}{\times \;10}^{-3}$	MC = Metal concentration in samples (mg/kg) FI = Ingestion rate (mg/day/person) EFr = The exposure frequency (days/year) ED = Total exposure duration (years) RfD = Oral reference dose (mg/kg/day) Bw = Body weight for children and adults (kg) AT = The non-carcinogen averaging time (ED ×365 days/year)
Cancer Risk (CR)	ADI × SF	ADI = Average Daily Intake (mg/kg·day) SF = Cancer Slope Factor (mg/kg·day)^−1^
Target Cancer Risk (TCR)	$\frac{(\mathrm{Cb}\;\times \;I\;\times \;10^{-3\;}\times \;\mathrm{CPSO}\;\times \;\mathrm{EFr}\;\times \;\mathrm{EDtot})}{\left(\mathrm{BWa}\;\times \;\mathrm{ATc}\right)}$	Cb = Heavy metal concentrations in samples (mg/kg) I = The ingestion rate (mg/day/person) CPSO = The carcinogenic potency slope, oral (mg/g/day)^−1^ EFr = The exposure frequency (days/year) EDtot = Total exposure duration (years) ATc = The carcinogen averaging time (days/year) ED × 365

### 5.2. Non-Carcinogenic Risk Assessment

#### 5.2.1. Health Risk Index (HRI)

In determining the HRI value, the equation shown in Table 4 involves the ratio between the daily intake of metal through consumption and the oral reference dose, together with general body weight of children and adults [12,106,148,149,150,151]. An HRI index value of more than 1 shows that human health is at risk or unsafe [152]. A changing of age in adults and children has determined the observation of health risk. The exposure was observed differently because of the different contact pathway. The health risk between age groups and locality of inhabitants may not be similar. [153]. Table 5 shows non-carcinogenic risk assessment from rice consumption in selected areas of different countries. The findings by Satpathy et al. [32] found that the Zn element has the highest HRI value in adults, which is beyond the value of 1. Similar to Neeratanaphan et al. [95], besides Pb, the HRI value for Mn was found to be greater than 1. However, Zn and Mn are known to be essential micronutrients that are necessary for plant growth; however, the micronutrients can be extremely toxic at high concentrations [154]. Although the health risk of single metal exposure was generally considered to be safe for consumption, the combination of several heavy metals may cause risk to local inhabitants. The exposure to two or more pollutants may result in additive or interactive effects [155].

#### 5.2.2. Hazard Quotient (HQ) and Target Hazard Quotient (THQ)

HQ and HI are example models that are used to analyze human non-carcinogenic risk for local inhabitants. Besides using total HRI value, the hazard quotient (HQ) also can be used to determine HI. The HQ formula that is shown in Table 4 is almost similar to HRI, where the equation involves the ratio of average daily intake (ADI) and reference dose (RfD) [147]. This equation is able to characterize the health risk of non-carcinogenic adverse effects due to exposure to toxicants [156,157], by which RfD is the estimated allowable dose for human via daily exposure [37]. The target hazard quotient (THQ) is another term used other than HQ to measure the potential non-carcinogenic effects of individual heavy metal [158]. It is used as a method to estimate risk, as provided by USEPA [158] in the U.S EPA Region III risk-based concentration table and the equation involved was based on Chien et al. [159], Yang et al. [160], and Fang et al. [161], as shown in Table 4. If HQ or THQ < 1, adverse health effects would unlikely be experienced, whereas, if HQ or THQ ≥ 1, then potential non-carcinogenic effects would occur [37,162,163].

Fan et al. [143] and Zeng et al. [37] reported that the HQ levels of Cd via brown rice consumption for local residents in Hunan Province exceeded 1, which indicated that this could pose potential non-carcinogenic risks for human health and are likely to increase due to the continuing consumption of contaminated brown rice by resident near mines area. Similarly, Wang et al [164] has revealed that the mean value of THQ for Cd exceeded the threshold value. The value indicates that the local inhabitants carry high potential for chronic health risk through rice consumption.

In Nigeria, HQ for Cd and Pb was found > 1, which will likely induce adverse health effect via rice consumption [146]. In contrast, Horiguchi et al. [165] suggested that heavy metals that are ingested by human are not equal to the absorbed pollutant dose in reality, as some fraction from the ingested heavy metals may be excreted and some of them may remain accumulated in the human body tissues, which could possibly affect their health. Meanwhile, in Iran, Djahed et al. [137] discovered that As has a HQ value more than 1, which indicated a considerable non-carcinogenic adverse health effect and consumption of rice from the collected samples is probably unsafe.

#### 5.2.3. Hazard Index (HI)

HI is calculated through the average daily consumption of rice for individual (adults and children) in order to obtain the measure of the potential risk of adverse health effects from a combination of chemical elements in rice. HRI or HQ can be summed across the constituents, as shown in Table 5, by assuming additive effects in order to calculate the hazard index (HI) for a specific receptor combination, such as diet [32,37,122]. Chronic risks are assumed to be unlikely to happen if the value of HI < 1, but, in case of the HI value reaching ≥ 1, non-cancer risk is likely to occur [143,166].

With reference to Table 5, Satpathy et al. [32] reported the HI values of heavy metals to be 1.561 and 1.360, respectively, for adults and children, in the East Coast Road, India, which indicates that they will undergo poor health effects in the near future due to the biomagnification caused by heavy metal accumulation over a period of time. Song et al. [41] stated the HI values of rice and vegetables in functional site of three toxic metals (As, Pb, and Cd) were greater than 1, whereas the HI values in the control site were less than 1 for adults and children. However, if the pollution continues to be severe in the control site, then it may greatly affect human health risks in their residents’ area.

In contrast with China, Zeng et al. [37] found the greater value of HI, which was up to 14.6 after evaluating the brown rice consumption with seven elements (Cd, Cr, As, Ni, Pb, Mn, and Hg). This value means that there is high non-carcinogenic risk from the ingestion of local brown rice. Praveena and Omar [167] found that rice that was collected from markets in Malaysia has a high potential of non-carcinogenic risk based on HI values for both adults and children by 27.0 and 18.0, respectively, through a combination of trace element and heavy metal exposure. Silins and Hogberg [168] suggested that cumulative trace element and heavy metal exposures will increase the health risks more than individual exposure of trace element and heavy metals.

**Table 5:** Non-carcinogenic risk assessment from rice consumption in selected areas of different countries.

Area of Study	HRI/HQ/THQ	Individuals	Risk Values						HI	References
			As	Pb	Cd	Cu	Cr	Zn		
East Coast Road, India	HRI	Adults	-	0.269	0.042	0.001	0.123	1.126	1.561	Satpathy et al. [32]
		Children	-	0.234	0.036	0.001	0.108	0.981	1.360	
Kalasin Province, Thailand	HRI	Local inhabitants	-	1.50	-	-	0.30	-	-	Neeratanaphan et al. [95]
Hunan Province, China	HQ	Local inhabitants	8.18	0.045	2.29	-	0.258	-	14.6	Zeng et al. [37]
Hunan Province, China	HQ	Local inhabitants	0.7264	0.0484	11.798	-	-	-	-	Fan et al. [143]
Fuzhou, China	THQ	Adults	0.8	0.1	0.6	0.3	0.00044	-	1.9	Fu et al. [169]
		Children	0.8	0.1	0.6	0.3	0.00050	-	2.0	
Zhejiang, China	HRI	Adults	0.34	0.84	0.77	-	-	-	-	Huang et al. [46]
		Children	0.44	1.09	1.00	-	-	-	-	
Hunan Province, China	THQ	Local inhabitants	-	0.081	3.047	0.877	0.005	0.771	8.138	Wang et al. [164]
Enugu, Nigeria	HQ	Adults	-	1.11	1.20	-	0.008	0.24	3.028	Ihedioha et al. [145]
Malaysia	HQ	Adults	0.51	0.051	0.47	0.4	0.0008	0.26	27.0	Praveena and Omar [167]
		Children	0.33	0.11	0.3	0.25	0.005	0.17	18.0	
Iranshahr, Iran	HQ	Local inhabitants	5.23	0.14	0.15	0.32	-	-	1.64	Djahed et al. [137]

Note: Health Risk Index, HRI; Hazard Quotient, HQ; Target Hazard Quotient, THQ; Hazard Index, HI.

### 5.3. Carcinogenic Risk Assessment

#### Cancer Risk (CR) and Total Cancer Risk (TCR)

For carcinogenic risk assessment, cancer risk (CR) or target cancer risk (TCR) are calculated using the equation that is shown in Table 6 in order to estimate the incremental probability of an individual developing cancer over a lifetime. For example, a CR of 10^−4^ indicates a probability of 1 in 10,000 individuals developing cancer [170]. The total CR (CRt) from all carcinogens is summed by assuming the additive effects, if the multiple carcinogenic elements are present. USEPA Region III Risk-Based Concentration provided the method of estimating TCR [156]. According to Ma et al. [171] and Cao et al. [166], it is considered acceptable if the risks are in the range of 1.0 × 10^−6^ to 1.0 × 10^−4^.

The International Agency for Research on Cancer (IARC) [4] has categorized As, Cr, and Cd as carcinogenic to humans, whereas Pb, Co, Cu, Fe, Al, and Zn are non-carcinogenic to humans. A study conducted by Fu et al. [169] on the carcinogenic risk of As in rice among Fuzhou population, Jiangxi Province of China, found that the TCR values were slightly higher than the acceptable range, which are 3.5 × 10^−4^ for adults and 3.8 × 10^−4^ for children. The values showed that As was present in the form of inorganic As in rice with 100% bioaccessibility for consumers. Therefore, the authors concluded that the carcinogenic risk of As might be overestimated, since the percentage of inorganic As is not 100% in food commodities. The consumption of local brown rice by people in Hunan Province, China poses potentially great carcinogenic risk based on the multiple carcinogenic evaluations, which was up to more than 400× higher than the limit set by USEPA [156], which is one to one hundred in a million chance of additional human cancer over a 70-year lifetime [37].

Table 6 shows the summary of carcinogenic risk assessment via rice consumption in selected areas of different countries. With reference to the table, the CR value for Cd in brown rice grown near the three mining areas of Hunan Province was the highest, with a total cancer risk of 0.0423 in 2015 [37] and 0.1773 in 2017 [143], indicating a great potential of carcinogenic risk from brown rice consumption in this region due to metal mining and waste discharge. For Malaysia, a study on 22 varieties of marketed rice samples to determine both the total and bioaccessibility of heavy metals revealed that the cumulative carcinogenic health risk via rice consumption for the combined exposure of As and Pb in adult and children was 0.0049 and 0.0032, respectively [167]. The findings showed that the exposure from combined heavy metals has a potential carcinogenic risk, especially in children growth development. Zeng et al. [37] and Fan et al. [143] reported that Cd contribute to cancer risk by approximately 99.77% and 81.2%, respectively. Long-term exposure towards Cd was reported to lead to an increased risk of mortality from all cancers [172]. Recently, Djahed et al. [137] and Fakhri et al. [173] conducted a study in Iran on the carcinogenic risk assessment from rice consumption among local inhabitants and found that the ingestion of rice for lifetime consumption would induce cancer risk, since the values of CR for both studies exceed the acceptable range. Hence, the remediation of contaminated soil is one of the effective measures to secure and reduce the risk of the local communities. In addition, it is strongly suggested that a routine monitoring of heavy metals in soil and rice in these areas be implemented.

**Table 6:** Carcinogenic risk assessment via rice consumption in selected areas of different countries.

Area of Study	CR/TCR	Individuals		Heavy Metals				CRt	References
			As	Pb	Cd	Ni	Cr		
Hunan Province, China	CR	Local inhabitants	0.00368	-	0.0343	0.00393	0.000388	0.0423	Zeng et al. [37]
Hunan Province, China	CR	Local inhabitants	0.0003	-	0.1769	-	-	0.1773	Fan et al. [143]
Fuzhou, China	TCR	Adult	0.00035	-	-	-	-	NA	Fu et al. [169]
		Children	0.00038	-	-	-	-	NA	
Malaysia	CR	Adult	>0.0001	<0.0001	-		-	0.0049	Praveena and Omar [167]
		Children	>0.0001	<0.0001	-	-	-	0.0032	
Iranshahr, Iran	CR	Local inhabitants	0.00237	-	-	-	-	NA	Djahed et al. [137]
Iran	CR	Local inhabitants	0.04864	0.02623	-	-	-	0.0749	Fakhri et al. [173]

Note: Cancer risk, CR; Target Cancer Risk, TCR; Total Cancer Risk, CRt; Not available, NA.

### 5.4. In Vitro Digestion Model

Other than estimating HRA by mathematical models, *in vitro* digestion models can be conducted in order to determine the bioaccessible form of trace element and heavy metals [174,175]. This model involves the total fraction of trace element and heavy metal concentration present in a specific environmental field within a time period and uptake through the indirect ingestion by organisms [176]. Rijksinstituut voor Volksgezondheid en Milieu (RIVM) in vitro digestion model is an example of the bioavailability method used in rice studies via rice ingestion, where the models involved are quite similar to the physiological conditions in a human body. According to Fernández-García et al. [177], the *in vitro* model minimizes the use of experimental animals and the massive number of different matrices, which makes it easier to measure a large number of sample and allows for replication.

These kinds of characteristics make it widely applicable for health assessment and nutritional efficiency prediction. There are three compartments that are involved based on the human physiological condition, which are oral cavity, stomach, and small intestine, including the parameters of pH, residence time, and particle size [174]. According to Lee et al. [178], the bioavailability concentrations are much preferred when compared to total heavy metal concentration due to overestimates of human health risks from heavy metal exposure. However, it is insufficient to identify health risks that are posed by humans through consumption by only assessing the bioavailability in rice below the maximum permitted levels. The detailed HRA should be associated with both carcinogenic and non-carcinogenic health risks to understand heavy metal exposure among adults and children [167].

Cooked rice is considered to be the best form of matrix to be used in human health risks studies when compared to raw rice, as the sample must be as if it was ingested by consumers in a way to reflect real situation of human exposure [179]. Omar et al. [174] found that the bioavailability of heavy metal concentration in cooked rice samples was reduced from Zn > Fe > Cu > Cr > Cd, and all of the varieties of cooked rice samples studied were considered to be safe for human consumption. However, the concentration of harmful heavy metals, such as Cd and Cr in low amounts, can potentially risk renal impairment and bone disease, such as osteoporosis in adults, both male and female [51,180]. A study conducted by Yang et al. [181] on Cd bioaccessibility in uncooked rice from rural mining areas revealed that the bioavailability fraction of Cd in uncooked rice has a significant positive correlation with the total concentration of Cd in cooked rice. The bioaccessibility of Cd from food depends not only on its binding forms, but also on the properties of food, such as the source of food and processing method. A recent study by Praveena and Omar [167] reported that the bioaccessibility of trace element and heavy metal concentration in cooked rice samples are below the maximum permitted level set by Malaysian Food Regulation [182] with a decreasing order of Zn (4.3 mg/kg) > Fe (1.9 mg/kg) > Cu (1.1 mg/kg) > Al (0.89 mg/kg) > Cr (0.11 mg/kg) > Co (0.032 mg/kg) > Cd (0.027 mg/kg) > Pb (0.022 mg/kg) > As (0.016 mg/kg).

Furthermore, Praveena and Omar [167] revealed that no potential non-carcinogenic risk was found to exist in adults and children through individual trace element and heavy metal exposure (HQ <1), but As was found to be present as a potential carcinogenic health risk with a CR value of more than 1 × 10^−4^. Examples of potential health risk from As exposure from cooked rice grain are malignant neoplasms, melanosis, and depigmentation; hence, concern on children should be more focused, since As and Pb can affect their brain and nervous system development [183]. Limitations on the available data for Malaysian ingestion rate (IR) values may influence the HRA, since the study was based on a previous report by Zheng et al. [87].

## 6. Mitigation Methods

There are several preventive measures for reducing heavy metals exposure to human (Table 7), by reducing their concentration in soil, and finally reducing their rice uptake. These approaches can be categorized as: (i) agriculture management practices, (a) water management [184], (b) soil amendments [185,186], (c) nutrient management [187], and (d) Tillage management [188], (ii) Bioremediation strategies, (a) Phytoremediation [189], (b) Microbial remediation [190], and (iii) Genetic approaches [191].

**Table 7:** Mitigation methods for reducing heavy metals availability in rice grains.

Mitigation Methods	Heavy Metals	References
**Agriculture management practices**		
Alternate wet and dry method (AWD) converts As (III) to As (V) which is less soluble in water; henceforth less uptake by plants	As(III), As(V)	Rinklebe et al. [192]
(a) Water management		
Flooding before and after heading lessens Cd concentration while aerobic condition rises Cd concentration in rice	Cd	Hu et al. [193]
AWD states reduced the activity of Hg(II)-methylating microbes which caused limited MeHg and THg concentrations in rice	MeHg, THg	Tanner et al. [194]
Silica strives with As(III) throughout uptake and down regulates Si transporters in root	As(III)	Wu et al. [195]
Application of silica limited Cd uptake and its accumulation in rice plants	Cd	Nwugo and Huerta [196]
Si and nano Si application limited Pb uptake in rice grain	Pb	Liu et al. [197]
Application of iron halt soluble Cd and As via formation of Fe plaques on root surface	Cd, As	Suriyagoda et al. [198]
(b) Soil amendments		
Fly Ash addition and Steel Slag lessens the Pb and Cd uptake in rice owing to immobilization of heavy metals in soil via *in situ*	Pb, Cd	Gu et al. [199]
Reduction of Cd solubility in soil as a result of their high calcium content, total calcium carbonates and alkalinity.	Cd	Shaheen and Rinklebe [200]
(c) Nutrient management		
Sulphur boosts complexation of As by synthesising Fe plaques and forming thiols	As	Dixit et al. [190]
Application of sulphur declines Cd and As uptake due to the rise of glutathione contents in the plants leaves	Cd, As	Fan et al. [201]
Application of phosphorus rises soil pH leading to sorption of Cd in the soil	Cd	Ahn et al. [202]
PO_4_^3−^ ions changed into Pb_5_(PO_4_)_3_OH when reacting with surface-adsorbed Pb.	Pb	Cao et al. [203]
Biochar adsorbs As and make it not available to plants	As	Yu et al. [204]
Biochar comprises of limestone (carbonate), which elevates soil pH, which encourages Cd precipitation and Cd sorption	Cd	Bian et al. [205]
Biochar retain Pb in soil as a result of high pH, cation exchange capacity (CEC), active functional groups, and porosity	Pb	Bian et al. [205]
(d) Tillage management		
High soil organic condition underneath reduced tillage management can elevate Cd adsorption and complexation	Cd	Gao et al. [206]
**Bioremediation strategies**		
Plants such as *Solanum nigrum*, *Arabidopsis halleri*, and many others, are classified as Cd hyperaccumulators and meant for phytoremediation of Cd contaminated sites.	Cd	Seregin et al. [207]; Wei et al. [208]; Ali et al. [209]
(a) Phytoremediation		
Some Azolla like species including *Azolla fi liculoides* Lam and *Azolla caroliniana* Willd. accumulates As in their body parts Chinese brake fern known as *Pteris vittata* L. is a famous As hyperaccumulator	As	Zhang et al. [210] Ma et al. [90]
Arbuscular mycorrhizal fungi (AMF) lessen uptake of Cd by rice, via changing chemical forms and subcellular distribution of Cd in rice	Cd	Li et al. [211]
Inoculation of rice with single or combined AMF reduced uptake of As in rice	As	Chan et al. [212]
(b) Microbial remediation		
*Sarchosphaera coronaria*, the ectomychorrizal fungus accumulates As at high levels in its fruiting bodies.	As	Falandysz and Borovička [213]
**Genetic approaches**		
*Paracoccus* species (*aioA* gene), the As (III) oxidizing- bacteria like converts As (III) to As (V) which is less mobile and less toxic than As (III). Inoculation of this species not just lessens the burden of As nonetheless encourages rice growth.	As(III), As(V)	Zhang et al. [214]
An *arsM* gene transforms As (III) into methylated species i.e., monomethylarsonic acid (MMA).	As(III)	Suriyagoda et al. [215]

## 7. Conclusions

Most rice producing countries experience heavy metal contamination, owing to human activities such as mining and smelting. Soils are the major sources of heavy metals, and its accumulation in grain is significantly important, as it will be processed for human consumption. Therefore, the health risk assessment on heavy metal exposure can provide an estimation of health risks that would be experienced by the targeted population, including infants and children. In fact, a standard of toxic heavy metal level in infant and children food products should be strictly imposed, as they are more susceptible to toxic metal poisoning. Data on bioavailability of heavy metals in rice grain are still insufficient, especially in high-risk areas, such as China and Japan. Besides, the availability data of the risk factor, such as ingestion rate in local area, should be of concern by relevant authorities for a particular population in a risk exposure assessment. Overall, the extensive study on heavy metal distribution should be done on risk areas of rice cultivation in order to reduce the public health risks and control rice yield for future demand.

## Figures and Tables

**Table 2:** Summary of the bioaccumulation factor (BAF) values in paddy plants from different areas.

Area of Study	Bioaccumulation Factor (BAF) Values								References
	As	Cd	Pb	Cr	Cu	Zn	Mn	Hg	
Dabaoshan mine, South China	-	0.20	0.005	-	0.013	-	-	-	Zhuang et al. [94]
Jiangsu Province, China	0.025	0.178	0.005	0.006	0.196	0.258	-	0.047	Hang et al. [12]
Ramgarh Lake, India (Control site)	0.014	0.016	0.03	0.002	0.002	0.007	0.038	0.272	Singh et al. [28]
Ramgarh Lake, India (Experimental site)	0.016	0.017	0.028	0.001	0.002	0.008	0.032	0.308	
East Coast Road (ECR), India	-	0.05–0.20	0.001–0.60	0.04–0.07	0.04–0.10	0.20–0.50	0.10–0.20	-	Satpathy et al. [32]
Kompipinan Papar district, Sabah	-	4.12	1.28	4.00	1.03	5.16	-	-	Payus et al. [53]
Ranau Valley, Sabah	0.24–0.89	-	-	0.00–0.01	0.00–0.03	0.07–0.10	0.01–0.02	-	Aziz et al. [52]
Isfahan Province, Iran	-	0.50–1.80	0.15–0.20	-	-	0.20–0.50	-	-	Rahimi et al. [99]
Kalasin Province, Thailand	-	-	0.23	0.10	-	-	1.88	-	Neeratanaphan et al. [95]

Note: -, Not included in analysis.

**Table 3:** Summary of the translocation factor (TF) values in paddy plants from different areas.

Area of Study	Paddy Parts	Translocation Factor (TF) Values								References
		As	Cd	Pb	Cr	Cu	Zn	Mn	Hg	
Ramgarh Lake, India (Control site)	Soil to root	4.19	11.34	0.39	0.04	0.07	0.02	0.16	0.96	Singh et al. [28]
	Root to shoot	0.04	0.08	0.22	0.33	0.07	0.41	0.43	0.71	
	Shoot to grain	0.06	0.01	0.33	0.112	0.38	0.55	0.116	0.95	
Ramgarh Lake, India (Experimental site)	Soil to root	4.20	7.19	0.38	0.05	0.09	0.03	0.18	1.28	
	Root to shoot	0.03	0.10	0.26	0.27	0.08	0.34	1.03	0.33	
	Shoot to grain	0.09	0.02	0.28	0.10	0.29	0.75	0.54	0.71	
East Coast Road (ECR), India	Soil to root	-	0.30–0.60	0.20–0.40	0.20–0.30	0.09–0.20	0.40–0.90	0.30–0.70	-	Satpathy et al. [32]
	Root to shoot	-	1.30–2.40	0.07–0.30	0.50–0.80	0.20–0.60	0.20–0.50	1.30–2.30	-	
	Shoot to grain	-	0.09–0.20	0.04–0.80	0.30–0.70	1.10–2.50	1.00–1.50	0.20–0.30	-	
Ranau Valley, Sabah, Malaysia	Roots to shoots	-	-	-	0.03–0.06	0.04–0.34	2.01–2.48	0.58–1.28	-	Aziz et al. [52]
Kompipinan Papar district, Sabah	Roots to shoots	-	0.94	0.29	1.97	0.37	3.43	-	-	Payus et al. [53]
**Area of Study**	Paddy parts	Translocation Factor (TF) Values								References
		**As**	**Cd**	**Pb**	**Cr**	**Cu**	**Zn**	**Mn**	**Hg**	
Isfahan Province, Iran	Soil to root	-	1.52	0.486	-	-	0.389	-	-	Rahimi et al. [99]
	Root to shoot	-	0.688	0.656	-	-	0.732	-	-	
	Shoot to grain	-	0.854	0.456	-	-	1.228	-	-	
Kalasin Province, Thailand	Soil to root	-	-	2.10	0.28	-	2.76	-	-	Neeratanaphan et al. [95]
	Root to stem	-	-	0.11	0.49	-	3.80	-	-	
	Stem to leaf	-	-	3.23	1.40	-	1.90	-	-	
	Stem to grain	-	-	1.67	1.14	-	0.22	-	-	

Note: -, Not included in study.

## Data Availability

Not applicable.

## Abbreviations

ADI	acceptable daily intake
AL	aluminum;
AMF	arbuscular mycorrhizal fungi
As	arsenic
BAF	bioaccumulation factor
BW	body weight
Cd	cadmium
CDI	chronic daily intake
cm	centimeter
Co	cobalt
COOH	carboxylic acid group
Cr	chromium
CR	cancer risk
Cu	copper
DIs	daily intake
EC	European Commission
EF	enrichment factor;
EFSA	European Food Safety Authority;
FAO	Food and Agriculture Organization
FAOSTAT	Food and Agriculture Organization Statistics
FDA	US Food and Drug Administration
Fe	iron
GRiSP	Global Rice Science Partnership
Hg	mercury
HI	hazard index
HM	heavy metal
HQ	hazard quotient
HRA	health risk assessment
HRI	health risk index
IARC	International Agency for Research on Cancer
IR	ingestion rate
IRRI	International Rice Research Institute
JECFA	Joint Expert Committee on Food Additives
MMA	monomethylarsonic acid
MMT	million metric tons
Mn	manganese
Ni	nickel
Pb	lead
ppb	parts per billion;
RfD	oral reference dose
RIVM	Rijksinstituut voor Volksgezondheid en Milieu
Sb	antimony
TCR	target cancer risk
TDI	tolerable daily intake
TF	translocation factor
THQ	target hazard quotient
USEPA	US Environmental Protection Agency
WHO	World Health Organization
Zn	zinc
